# Medical management of septic arthritis of sternoclavicular joint: A case report

**DOI:** 10.1097/MD.0000000000022938

**Published:** 2020-10-30

**Authors:** Hea Yoon Kwon, Boram Cha, Jae Hyoung Im, Ji Hyeon Baek, Jin-Soo Lee

**Affiliations:** Department of Internal Medicine, Inha University School of Medicine, Jung-gu, Incheon, Republic of Korea.

**Keywords:** sternoclavicular joint, *Streptococcus agalactiae*, osteomyelitis

## Abstract

**Rationale:**

Sternoclavicular joint septic arthritis is an unusual disease in healthy adults, and *Staphylococcus aureus* is the most common causative pathogen. The current treatment of choice is surgery with sternoclavicular joint resection and pectoralis flap closure, especially when the disease is complicated by osteomyelitis and abscess.

**Patient concerns:**

Here, we report a 76-year-old woman without risk factors who visited our hospital for pain and redness, swelling on the left anterior chest wall.

**Diagnosis:**

Magnetic resonance imaging showed infectious arthritis in the left SCJ, with multiple abscess pockets at the subcutaneous layer of anterior chest wall communicating with the joint cavity. *Streptococcus agalactiae* was isolated from blood culture.

**Intervention:**

She was treated with 6 weeks of antibiotic therapy.

**Outcomes:**

After antibiotic treatment, she was successfully treated without recurrence.

**Lessons:**

Besides surgery, medical treatment should also be considered for sternoclavicular joint septic arthritis, depending on patient status and the causative pathogen. Physicians should be aware of this rare disease to facilitate its prompt diagnosis and management.

## 1 Introduction

The sternoclavicular joint (SCJ) is an unusual site of septic arthritis, accounting for 0.5% to 1.0% of all joint infections.^[1,2]^ The known risk factors for SCJ septic arthritis are intravenous drug use (21%), infection at a distant site (15%), diabetes mellitus (13%), trauma (12%), infected central line (9%), and chronic renal failure (8%).^[1,3]^ Cases of SCJ arthritis in healthy adults without risk factors have been reported.^[1,4]^

*Staphylococcus aureus* is the most common causative pathogen among culture-proven cases of SCJ septic arthritis (49%), followed by *Pseudomonas aeruginosa* (10%), *Brucella melitensis* (7%), and *Escherichia coli* (5%). ^[3,5]^ Other causative pathogens such as group B streptococcus, *Mycobacterium tuberculosis*, *Streptococcus pneumoniae*, and polymicrobial bacteria have also been reported.^[5,6]^ A rapid diagnosis of SCJ septic arthritis by imaging studies is important, as this condition could lead to serious complications such as osteomyelitis (55%), chest wall abscess or phlegmon (25%), and mediastinitis (13%).^[1,6–8]^ After the diagnosis of SCJ arthritis is established, surgical treatment with en-bloc resection of the SCJ and ipsilateral pectoralis major muscle flap is preferred in the definitive treatment approach.^[7]^ Conservative treatment with antibiotics, surgical drainage, and debridement has shown a failure rate of 83%.^[9]^ However, herein, we report a case of an old woman with SCJ septic arthritis caused by *S agalactiae* who was successfully treated solely with antibiotics.

## 2 Case presentation

**Figure 1:**
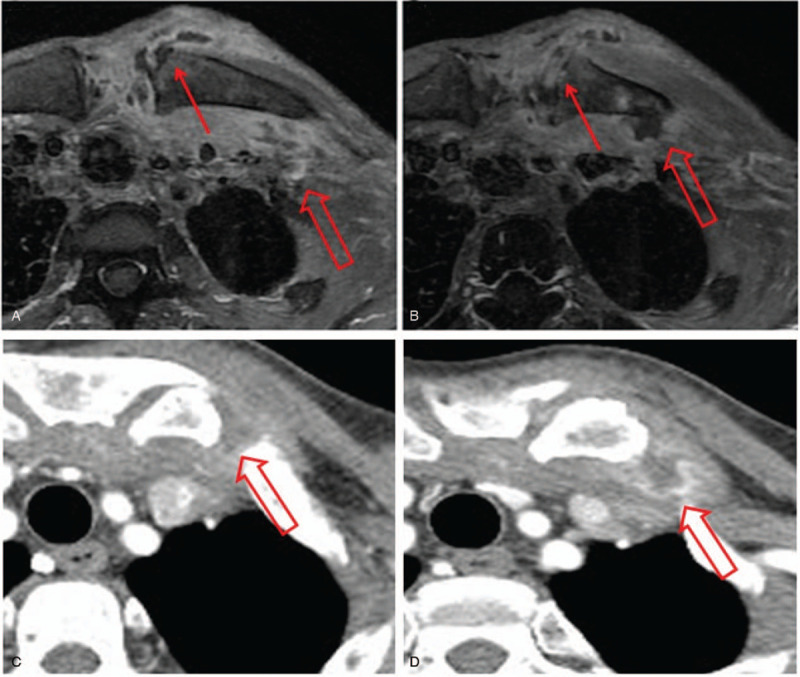
(A and B) Fat suppression phase of magnetic resonance imaging demonstrates left SCJ infectious arthritis with multiple small abscesses at the subcutaneous layer (arrow), and abscess pockets posterior to the clavicle (empty arrow). (C and D) Contrast enhanced chest computed tomography was performed after four weeks of antibiotic treatment. Decreased size of abscess pockets posterior to the clavicle (empty arrow) is observed.

A 76-year-old woman visited the Emergency Room complaining of swelling, redness, and warmth on the left anterior chest wall for 4 days. She had a medical history of cerebral infarction 15 years ago, and had since been taking aspirin and clopidogrel. She had been diagnosed with endometrial cancer and had undergone hysterectomy 8 years ago, which had been followed by chemotherapy and radiotherapy. Moreover, she had developed a lumbar spine tumor 7 years ago and had subsequently undergone radiotherapy, although she and her family were unaware of the pathological diagnosis of the tumor. Both her cancers were completely treated. Three months before visiting our hospital, she had been diagnosed with delirium and was taking risperidone and donepezil. She did not have history of diabetes mellitus or hypertension. Her estimated glomerular filtration rate was slightly decreased to 43.8 mL/min/1.73 m^2^. The patient appeared acutely ill. There was no cardiac murmur on auscultation. Her initial blood pressure was 107/56 mm Hg, heart rate was 96 beats per minute, respiratory rate was 24 breaths per minute, and body temperature was 38.1°C. Her white blood cell count was 6.5 × 10^9^/L (normal range: 4.0–10.0 × 10^9^/L), with 82.3% neutrophils (normal range: 40.0–75.0%); her C-reactive protein level (CRP) was 25.08 mg/dL (normal range: 0–0.5 mg/dL) and ESR was 127 mm/h (normal range: 1–22 mm/h). Physical examination revealed no abnormalities in her teeth or throat. Cardiac echocardiography reveled no vegetation or structural abnormalities on the valves. Magnetic resonance imaging showed infectious arthritis in the left SCJ, with multiple abscess pockets measuring 3.2 cm × 3 cm × 1 cm at the subcutaneous layer of anterior chest wall communicating with the joint cavity (Fig. 1A and B). In addition, small abscesses were observed posterior to the left SCJ and sternum, and posterior to the clavicle. Osteomyelitis was observed at the sternum, and erosions were observed in the clavicle. Ultrasound-guided aspiration of the joint fluid for culture was performed. Cefazolin was initially started, empirically targeting methicillin-susceptible *S aureus* (MSSA), which is the most common pathogen of SCJ septic arthritis.

**Figure 2:**
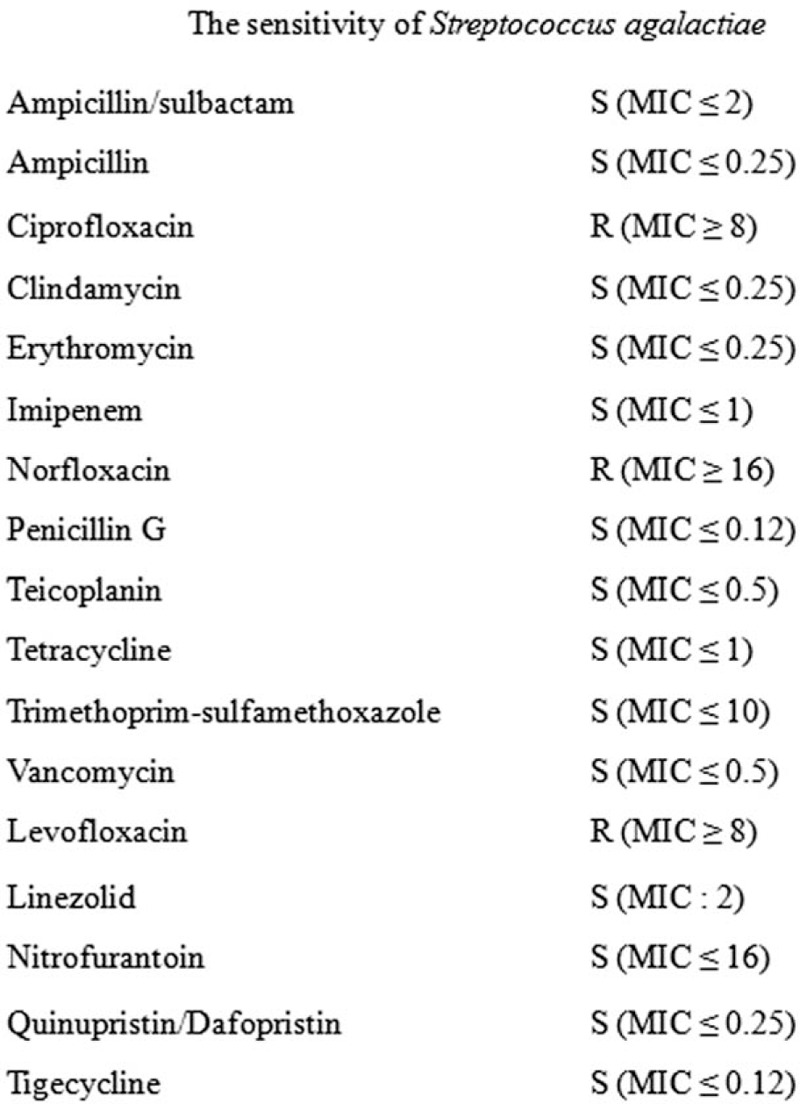
Sensitivity of *Streptococcus agalactiae*.

**Figure 3:**
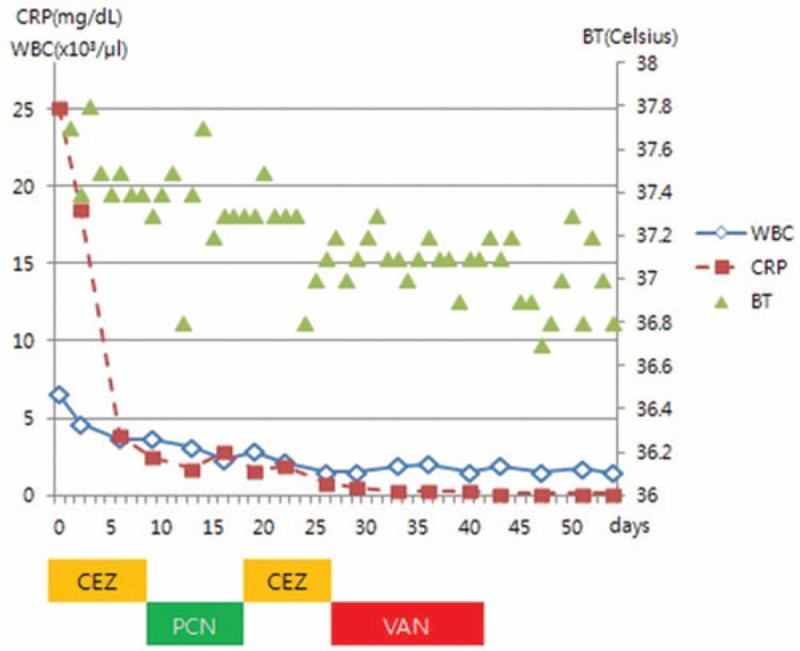
Timeline of the patient's course. Cefazoline was prescribed as 6 g/day; penicillin G potassium, as 24 million units/day; and vancomycin, as 1.5 g/day. BT = body temperature, CEZ = cefazolin, CRP = C-reactive protein, PCN = penicillin G potassium, VAN = vancomycin, WBC = white blood cells.

Penicillin-sensitive (minimum inhibitory concentration ≤0.12) *Streptococcus agalactiae* was isolated on both peripheral blood and aspirated joint fluid. The sensitivity of *S agalactiae* is shown in Figure 2. The antibiotic was switched to penicillin G 24 million units per day. After 2 weeks of treatment, the CRP level of the patient decreased from 25.08 to 1.67 mg/dL (Fig. 3). However, she developed leukopenia and thrombocytopenia, which were suspected to be the side effects of penicillin. Despite changing the antibiotic to cefazolin, her leukopenia worsened. Subsequently, cefazolin was replaced by vancomycin, and the treatment was continued for another 2 weeks. After a total treatment duration of 6 weeks, the CRP level of the patient decreased to the normal range (0.25 mg/dL), and follow-up computed tomography of the chest showed improved SCJ swelling and abscess (Fig. 1C and D). She was discharged, and she did not show any signs of recurrence after 3 months, after which she was lost to follow-up.

The daughter-in-law of the patient has provided informed consent for publication of the case (attached as supplement).

## 3 Conclusions

Although a 17% incidence of SCJ arthritis has been reported among intravenous drug users, this infection is relatively rare among the general population, with an estimated incidence of 1%.^[2,3,7]^ SCJ infection usually occurs in patients with predisposing risk factors such as intravenous drug use, hemodialysis, infected central venous line, diabetes mellitus, alcohol abuse, corticosteroid treatment, cancer, trauma, radiation therapy, and surgery.^[3]^ SCJ arthritis occurs in patients with contiguous or distant foci of infection, such as pneumonia, cellulitis, endocarditis, urosepsis, septic pulmonary emboli, epidural abscess, intra-abdominal abscess, gingivitis, and disseminated tuberculosis.^[3,10]^

Our case is unique in that our patient did not have any underlying disease except decreased glomerular filtration rate. Although SCJ septic arthritis typically affects immune-compromised patients or intravenous drug users, several cases of SCJ arthritis in previously healthy patients have been reported.^[1,6,11]^ Physicians should be aware of this disease when a patient complains of insidious onset of chest pain or pain in the ipsilateral neck and shoulder. A suspicion of this disease and prompt diagnosis for its management is important, as this disease is associated with serious complications such as osteomyelitis, chest wall abscess, and mediastinitis.^[10,12,13]^

Savcic-Kos et al presented a case of Group A streptococcal SCJ arthritis in an immune-competent patient,^[5]^ in which previous pharyngitis was the suspected infection focus. They suggested that minor trauma may facilitate bacteremic seeding in SCJ. Our patient had group B streptococcal SCJ arthritis; however, we failed to clarify the origin of the infection. As group B streptococcal bacteremia is rare in non-pregnant adults, it may occur without apparent focus or with soft tissue infection.^[14]^

Our case is noteworthy in that the patient was completely treated solely by antibiotics, although surgery is the main treatment for septic arthritis of SCJ in many centers. Song et al reported that complete SCJ resection and pectoralis flap closure did not result in recurrences among patients with SCJ septic arthritis, while debridement and antibiotic therapy alone were associated with recurrence in five out of six patients.^[9]^ Failure of successful treatment with conservative management was explained by poor capacity of the manubrium to clear the established infection, and the presence of widespread infectious involvement of the surrounding tissues owing to the chronic nature of SCJ arthritis.

However, the patient in this case was successfully treated by 6 weeks of antibiotic therapy, although she presented with multiple abscesses around the SCJ, and osteomyelitis. This case highlights that SCJ septic arthritis can be successfully treated not only by surgery but also with medicines, depending on the pathogen and its susceptibility to available antibiotics. Accurate diagnosis of the disease in the acute phase and eradication of the causative pathogen using antibiotics are important for successful medical treatment of SCJ septic arthritis.

In summary, we present SCJ septic arthritis in an immune-competent patient that was successfully treated solely with antibiotics. Physicians should be aware of this disease, as late diagnosis may result in various complications such as osteomyelitis, mediastinitis, and chest wall abscess.^[10,12,13]^ Besides surgery, which has been the preferred treatment for SCJ septic arthritis, medical treatment should also be considered, depending on the pathogen, immunity of the patient, and severity of the disease.

## Author contributions

**Conceptualization:** Jin-Soo Lee.

**Data curation:** Boram Cha.

**Formal analysis:** Boram Cha.

**Investigation:** Hea Yoon Kwon, Jae Hyoung Im.

**Resources:** Ji Hyeon Baek.

**Supervision:** Jin-Soo Lee.

**Validation:** Ji Hyeon Baek.

**Writing – original draft:** Hea Yoon Kwon.

**Writing – review & editing:** Hea Yoon Kwon.
